# Selective Oxidation of H_2_S to Elemental Sulfur over Waste PET-Derived Biochar-Supported Metal Catalysts

**DOI:** 10.1021/acsomega.5c08359

**Published:** 2025-12-08

**Authors:** Jia-Yin Lin, Guan-Jie Wang, Jia-Yu Lee, Yu-Lun Wu, Chih-Ying Wang

**Affiliations:** † Graduate Program in Semiconductor and Green Technology, Academy of Circular Economy, 34916 National Chung Hsing University, Taichung 402, Taiwan; ‡ Graduate Program in Industrial and Smart Technology, Academy of Circular Economy, National Chung Hsing University, Taichung 402, Taiwan; § Innovation and Development Center of Sustainable Agriculture, National Chung Hsing University, Taichung 402, Taiwan

## Abstract

The upcycling of plastic waste into high-value functional materials offers a sustainable pathway aligned with green chemistry and circular economy principles. In this study, activated carbon (aPAC) was synthesized from waste PET bottles via KOH activation and employed as a support for trivalent metal catalysts (Fe, Bi, and Ce) aimed at low-temperature catalytic oxidation of hydrogen sulfide (H_2_S). Among the prepared catalysts, Fe/aPAC exhibited the most promising performance, achieving nearly complete H_2_S conversion (∼95%) and a high sulfur yield (∼90%) under humid air at 30 °C. Textural and surface characterizations (X-ray diffraction, Fourier transform infrared, X-ray photoelectron spectroscopy, CO_2_-temperature-programmed desorption, and scanning electron microscopy [SEM]/EDS) revealed that the presence of Fe_2_O_3_/FeOOH, abundant oxygenated groups (C–O, C—O, O–H), and surface basicity collectively enhanced redox activity and H_2_S adsorption. Notably, the catalyst maintained over 80% conversion after three regeneration cycles, demonstrating excellent stability and reusability. Extracted sulfur products were confirmed via SEM and visible recovery. A reaction mechanism involving H_2_S dissociation, Fe^3+^/Fe^2+^ redox cycling, and surface-assisted O_2_ activation was proposed. This work not only provides mechanistic insights into H_2_S oxidation under mild conditions but also proposes a cost-effective and scalable approach for converting PET waste into efficient catalytic materials, offering dual environmental benefits in sulfur recovery and plastic valorization.

## Graphical abstract

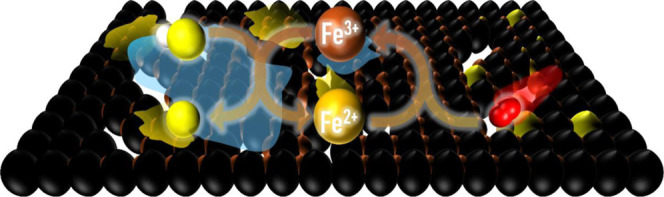

## 1 Introduction

Hydrogen sulfide (H_2_S) is a highly toxic, corrosive, and malodorous gas that poses significant risks to both human health and the environment.^1–3^ It is commonly found in biogas, natural gas, petroleum refining, wastewater treatment processes, and various industrial emissions.^4–6^ Even at low concentrations, H_2_S can cause respiratory distress and eye or mucous membrane irritation, while high concentrations can be lethal.^7^ Additionally, H_2_S accelerates the corrosion of metal equipment and infrastructure, leading to safety hazards and economic losses.^8,9^ Therefore, the effective control and removal of H_2_S are of great importance. Common strategies for H_2_S removal include absorption, adsorption, biological treatment, and catalytic oxidation.^10–13^ Wet scrubbing using alkaline or oxidative solutions is widely used in industry but often suffers from high chemical consumption and wastewater generation. Adsorption using activated carbon or metal oxides offers simplicity but is limited by the adsorption capacity and regeneration issues. Biological desulfurization is eco-friendly but sensitive to environmental conditions and slow in reaction rate.^10,14^ In contrast, catalytic oxidation is considered one of the most promising technologies due to its high efficiency,^15^ low energy requirements, and ability to selectively convert H_2_S into elemental sulfur (S^0^) under mild conditions. This not only avoids the formation of unwanted SO_2_ or SO_4_^2–^ byproducts but also enables the recycling of sulfur, thereby aligning with the principles of a circular economy. The catalytic oxidation of H_2_S generally involves the initial adsorption and activation of H_2_S molecules on the catalyst surface, followed by their reaction with activated oxygen species to produce elemental sulfur (S^0^) and water. The reaction under mild oxidative conditions can be simplified as eq 1

1 $$H_{2}S+1/2\;O_{2}\to S^{0}+H_{2}O$$

The key to this reaction lies in the ability of the catalyst to activate molecular oxygen (O_2_) and provide reactive oxygen species (ROS), such as O^–^, O_2_^–^, or lattice oxygen, while maintaining good selectivity toward elemental sulfur. On the other hand, the environmental threat posed by postconsumer PET (polyethylene terephthalate) bottles has grown increasingly severe.^16^ Although recycling PET into raw materials for remanufacturing is widely promoted, a significant portion of PET waste is still incinerated, resulting in resource loss and secondary pollution. Recent advances in thermochemical conversion have enabled the transformation of waste PET into porous carbon materials,^17,18^ which possess high surface area and tunable pore structures. When modified with suitable metal species, these carbon materials can serve as low-cost yet efficient catalyst supports for environmental applications.

Despite extensive research on biomass-derived carbon materials for H_2_S removal, most studies have focused on physical adsorption or simple metal oxide impregnation with limited investigation into the redox behavior and sulfur speciation under mild oxidation conditions. Recent efforts have incorporated CaCO_3_–ZnO loaded biochar,^19^ MgFe_2_O_4_/N-doped biochar,^20^ Cu-MOF-derived Cu on N-doped carbon,^21^ MgO–MnO loaded aerogels,^22^ and Mg-MOF derived MgO biochar composites,^23^ which demonstrate improved low-temperature desulfurization but often involve complex synthesis procedures, expensive precursors, or nitrogen doping to enhance activity.

In contrast, this study introduces a scalable, low-cost strategy using non-nitrogenous waste PET as the carbon source, modified via one-step impregnation with trivalent metal nitrates (Fe^3+^, Ce^3+^, Bi^3+^) to yield catalysts with enhanced basicity, redox capacity, and sulfur affinity. These metal species were selected based on their distinct catalytic roles: Fe^3+^ facilitates redox cycling, Ce^3+^/Ce^4+^ provides oxygen storage-release capability, and Bi^3+^ tunes surface acidity. To the best of our knowledge, this is the first report integrating PET-derived activated carbon with trivalent metal modifiers for selective H_2_S catalytic oxidation under ambient conditions. More importantly, this work not only achieves nearly complete H_2_S conversion at 30 °C but also elucidates the reaction mechanism through a combination of sulfur speciation, X-ray photoelectron spectroscopy (XPS), Fourier transform infrared (FTIR), and X-ray diffraction (XRD) analyses. Therefore, our approach addresses both plastic waste valorization and air pollutant control in a unified system, aligning with the principles of green chemistry and circular economy.

## 2 Materials and Methods

### 2.1 Reagents and Chemicals

Postconsumer PET bottles were collected from local sources near the institute. Cerium­(III) nitrate hexahydrate (Ce­(NO_3_)_3_·6H_2_O) was obtained from UNI-ONWARD (Taiwan), while bismuth­(III) nitrate pentahydrate (Bi­(NO_3_)_3_·5H_2_O, 98%) was purchased from Thermo Scientific (USA). Iron­(III) nitrate nonahydrate (Fe­(NO_3_)_3_·9H_2_O) was acquired from Scharlau (Spain). Potassium hydroxide (KOH) and sodium hydroxide (NaOH) were supplied by Emperor Chemical (Taiwan). All aqueous solutions were prepared by using either ultrapure water or deionized (DI) water, depending on experimental requirements.

### 2.2 Synthesis of M^3+^ Loaded Carbon Composites from Waste PET

The preparation process of the catalysts is illustrated in Figure 1. To obtain the carbon precursor, 4 g of waste PET was mixed with 8 g of KOH in 50 mL of DI water and stirred at ambient temperature for 16 h.^17,24,25^ The resulting mixture was dried at 80 °C, followed by carbonization in a tubular furnace under a nitrogen atmosphere. The heating rate was set to 10 °C/min with a final holding temperature of 800 °C for 2 h. The obtained black carbon product was thoroughly washed with DI water to remove residual alkali and then dried at 80 °C. This material was denoted as aPAC (KOH-activated porous carbon), while the control sample without KOH treatment was labeled PAC. For trivalent metal loading, 8 g of aPAC was impregnated with 2 g of Fe­(NO_3_)_3_·9H_2_O and stirred for 10 min at room temperature. Then, 56 g of NaOH was added to the suspension and stirred for another hour.^26–28^ The solid product was recovered by centrifugation, washed three times with DI water, and dried at 80 °C, yielding Fe/aPAC. The same procedure was applied using Ce­(NO_3_)_3_·6H_2_O and Bi­(NO_3_)_3_·5H_2_O to prepare Ce/aPAC and Bi/aPAC, respectively.^29^

**Figure 1:**
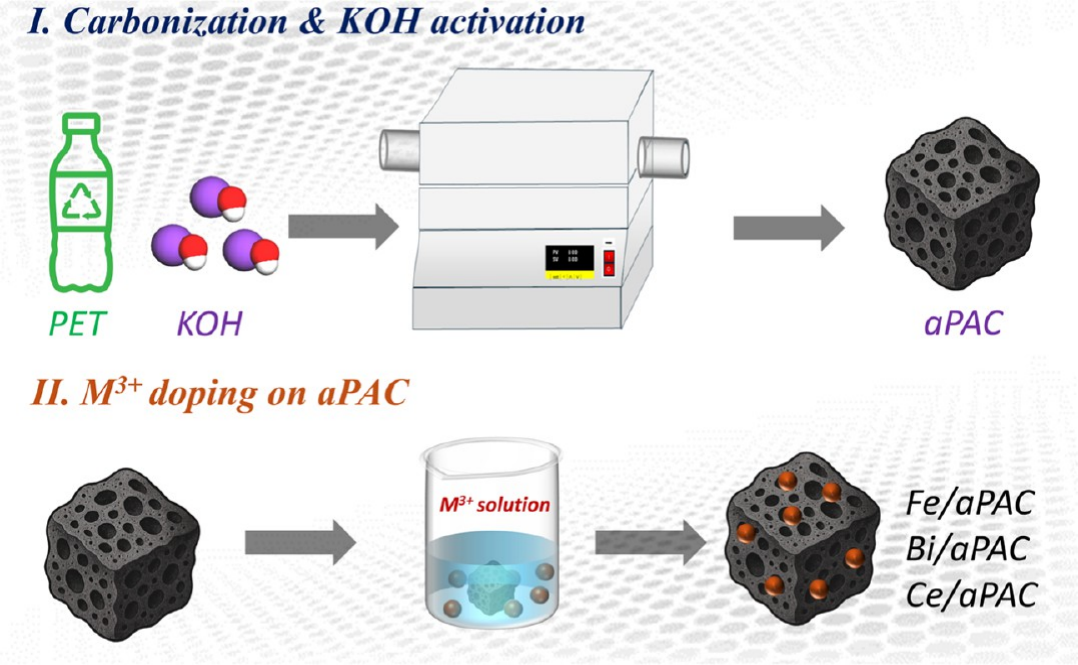
Schematic of catalyst preparation: (I) KOH-assisted carbonization of PET to form aPAC; (II) M^3+^ (Fe^3+^, Bi^3+^, Ce^3+^) doping to obtain Fe/aPAC, Bi/aPAC, and Ce/aPAC.

The amount of sulfur collected was calculated based on the difference between the inlet and outlet H_2_S concentrations over time, using the following equation^20,23^

2 $$sulfur\;collected\;\left(mg\;S/g\right)=32.07\times Q\times \frac{\int \left({\left[H_{2}S\right]}_{in}-{\left[H_{2}S\right]}_{out}\right)dt}{m}$$

where *Q* is the total gas flow rate (L min^–1^), ${\left[H_{2}S\right]}_{in}$ and ${\left[H_{2}S\right]}_{out}$ represent the inlet and outlet H_2_S concentrations, and 32.07 is the atomic weight of sulfur (g mol^–1^). *m* denotes the mass of the catalyst sample (g). This formula yields the total sulfur produced during the reaction, expressed in milligrams (mg), without normalization to the catalyst mass.

### 2.3 Characterization of M^3+^ Loaded Carbon Composites from Waste PET

The morphology and microstructure of the synthesized materials were investigated by using scanning electron microscopy (SEM, JEOL, Japan) and transmission electron microscopy (TEM, JEOL, Japan). Elemental distribution and surface composition of Fe, Ce, Bi, and S were examined by energy-dispersive X-ray spectroscopy (EDS). Crystalline phases were identified using XRD (Bruker, USA). Surface area and porosity were evaluated by nitrogen adsorption–desorption isotherms using a gas sorption analyzer (NOVAtouch, Anton Paar, Austria).

Thermogravimetric analysis (TGA; TGA 4000, PerkinElmer, USA) was conducted to assess the thermal stability. Chemical functionalities were analyzed by Fourier-transform infrared spectroscopy (FT-IR; iS5, Thermo Fisher Scientific, USA) in the range of 400–4000 cm^–1^. Surface chemical states were determined via X-ray photoelectron spectroscopy (XPS; PHI 5000, ULVAC-PHI, Japan), and Raman spectroscopy (MRID, ProTrusTech, Taiwan) was employed to analyze the carbon structure and defect levels. Surface basicity was estimated by CO_2_ temperature-programmed desorption (CO_2_-TPD; ASIQ TPx, Anton Paar, Austria), with detailed experimental procedures provided in the Supporting Information (S1). The procedure for extracting elemental sulfur from the used catalysts is also described in Supporting Information (S2).

### 2.4 Catalytic Oxidation of H_2_S Using M^3+^ Loaded Carbon Composites from Waste PET

Catalytic performance toward H_2_S oxidation was tested in a fixed-bed quartz tubular reactor equipped with an external heating system. Simulated gas mixtures were prepared using a gas mixer with nitrogen serving as the carrier and humidity control gas. A total of 0.2 g of catalyst was placed in the reactor, and the H_2_S-containing feed gas (100 ppm) was introduced at a gas hourly space velocity (GHSV) of 180,000 h^–1^. The outlet H_2_S concentration was continuously monitored using a portable gas analyzer (POLI, USA) to evaluate the removal efficiency under varying reaction conditions.

## 3 Results and Discussion

### 3.1 Characterization of As-prepared Catalysts

The surface morphology and microstructure of catalytic materials play crucial roles in determining their adsorption and catalytic performance, particularly in gas–solid heterogeneous reactions such as H_2_S oxidation. Surface area, porosity, metal dispersion, and nanoscale structure all influence the accessibility of active sites and the efficiency of reactive gas–solid interactions. Therefore, SEM and TEM were employed to investigate the morphology and structural features of the as-prepared catalysts, as shown in Figures 2 and 3. The SEM image of raw PET (Figure 2a) reveals a flat and sheet-like morphology characteristic of the polymeric substrate. After chemical activation and carbonization, the KOH-activated porous carbon (aPAC, Figure 2c) exhibits a rough and irregular surface, indicating successful carbon framework reconstruction.^15^ Compared with PAC, the sample prepared without KOH (PAC, Figure 2b) displays a denser and less textured morphology, highlighting the essential role of KOH in pore formation. Upon impregnation with trivalent metal nitrates, notable changes in surface texture are observed. The Fe-loaded sample (Fe/aPAC, Figure 2d) shows dense particle clusters uniformly distributed across the surface. EDS analysis confirms a high Fe content of 62.78 wt %, suggesting substantial metal deposition that may provide abundant catalytic sites but could also lead to partial agglomeration. The Bi/aPAC sample (Figure 2e) exhibits a granular morphology, and the Bi content reaches 75.69 wt %, indicating heavy surface loading. Such extensive deposition may alter the surface porosity and accessibility. In contrast, Ce/aPAC (Figure 2f) shows a more fibrous and open structure with 9.61 wt % Ce detected by EDS, suggesting better dispersion of Ce species, which may facilitate oxygen mobility and redox behavior.

**Figure 2:**
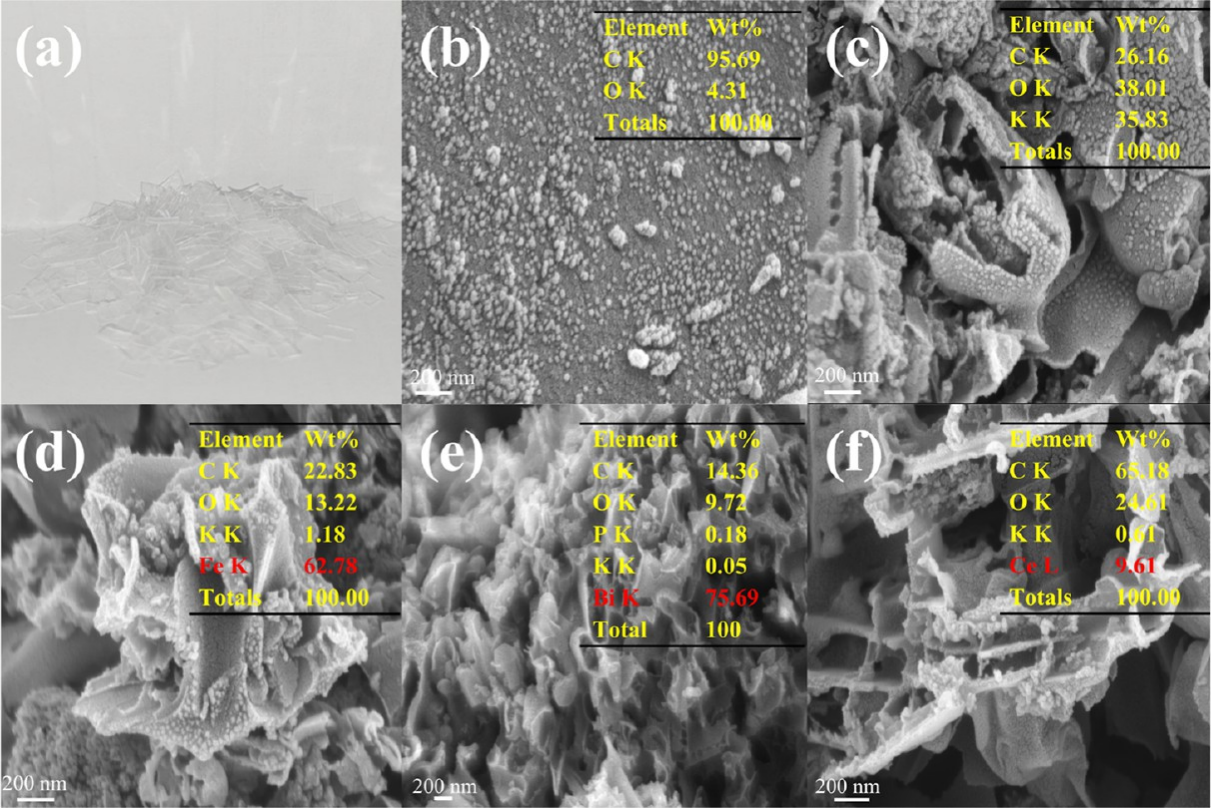
(a) Photograph of waste PET; SEM image of (b) PAC, (c) aPAC, (d) Fe/aPAC, (e) Bi/aPAC, (f) Ce/aPAC.

**Figure 3:**
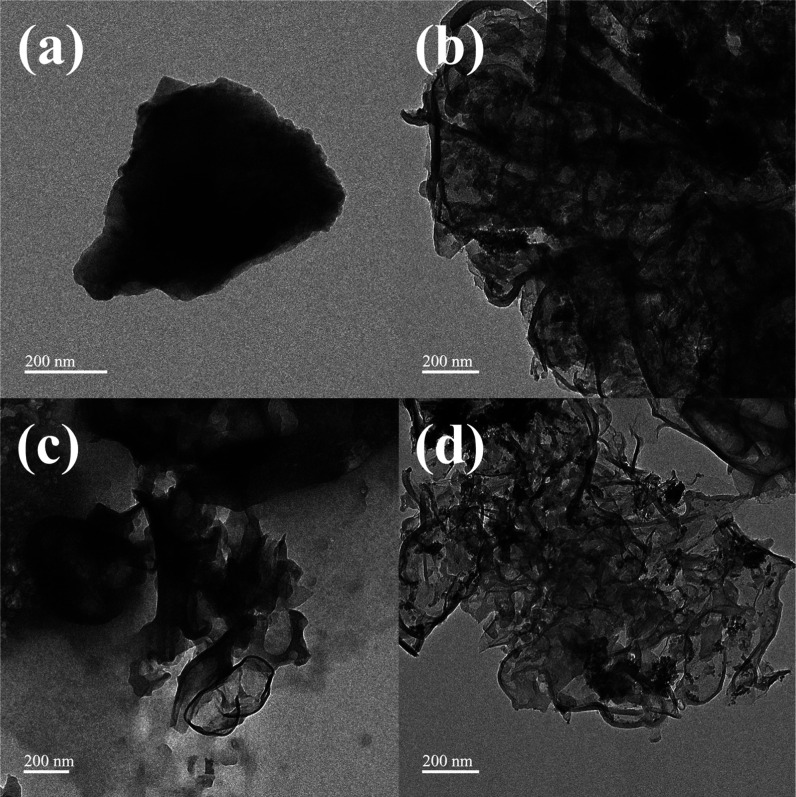
TEM image of (a) PAC, (b) Fe/aPAC, (c) Bi/aPAC, (d) Ce/aPAC.

Further insight into the microstructure was obtained through TEM analysis (Figure 3). PAC (Figure 3a) presents a dense and compact carbon matrix. In Fe/aPAC (Figure 3b), the formation of dark, irregular domains implies the presence of Fe-based nanoparticles or clusters. Both Bi/aPAC and Ce/aPAC (Figure 3c,d) display partially transparent, wrinkled carbon sheets, indicating the presence of disordered or amorphous metal oxides dispersed across the carbon support. The relatively loose and porous features observed in Ce/aPAC may contribute to enhanced gas diffusion and active site accessibility during the chemical reaction.

To understand the catalytic behavior of the synthesized materials, a comprehensive analysis of their structural, chemical, and textural properties was conducted. These characteristics are essential, as they directly influence the availability of active sites, gas adsorption–diffusion behavior, and ultimately the efficiency of H_2_S catalytic oxidation. XRD patterns (Figure 4a) first reveal the crystalline characteristics of the samples. Both PAC and aPAC display broad diffraction features centered around 2θ ≈ 25°, typical of amorphous carbon. In aPAC, several additional peaks are observed, corresponding to crystalline K_2_O_3_ (JCPDS 00-011-0655), indicating residual potassium from the activation process. In contrast, Fe/aPAC, Bi/aPAC, and Ce/aPAC show sharper peaks associated with crystalline metal oxides. Specifically, Bi/aPAC shows peaks assigned to Bi_2_O_3_ (JCPDS 00-027-0053),^30,31^ and Ce/aPAC exhibits reflections matching cubic CeO_2_ (JCPDS 03-065-5923).^32,33^ These results confirm the successful incorporation and in situ formation of metal oxide species on the activated carbon framework—an important structural basis for redox catalysis. No distinct peaks are observed for Fe/aPAC, suggesting that Fe species are highly dispersed or exist in an amorphous state.^34^

**Figure 4:**
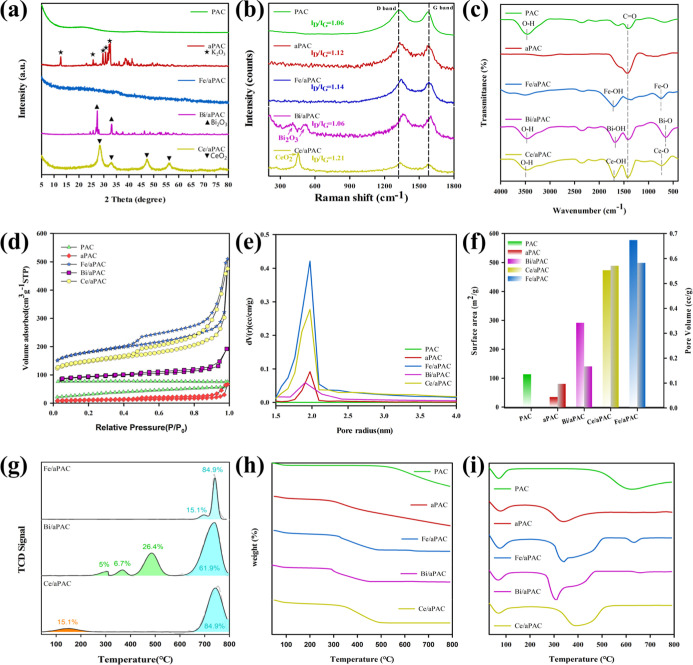
(a) XRD patterns, (b) Raman spectra, (c) FTIR spectra, (d) N_2_ adsorption–desorption isotherms, (e) BJH pore size distribution, (f) BET surface area and pore volume comparison, (g) CO_2_-TPD profiles, (h) TGA, and (i) DTG curves of PAC, aPAC, and metal-loaded aPAC catalysts (Fe/aPAC, Bi/aPAC, and Ce/aPAC).

Further insights into the carbon structure were obtained by Raman spectroscopy (Figure 4b). All samples show two prominent peaks near 1350 cm^–1^ (D band) and 1590 cm^–1^ (G band),^35^ corresponding to disordered and graphitic carbon, respectively. The intensity ratio (*I*_D_/*I*_G_), which reflects the degree of structural disorder, increases from PAC (0.86) to aPAC (1.12), and further to metal-loaded samples (up to 1.44).^36–38^ This suggests that both KOH activation and metal incorporation introduce more defects and edge sites, which are beneficial for enhancing the surface reactivity and metal anchoring. To complement the structural information, FTIR spectra (Figure 4c) were used to identify functional groups on the carbon surface. PAC and aPAC show characteristic peaks of the O–H, C–O, and C=O groups, while metal-loaded samples exhibit additional bands attributed to metal–oxygen bonds (e.g., Fe–O, Bi–O, and Ce–O), confirming the formation of metal oxide species. These surface functionalities can enhance H_2_S adsorption and play roles in redox interactions during catalytic oxidation.

Textural properties were then evaluated by N_2_ adsorption–desorption analysis. As shown in Figure 4d, all samples exhibit typical type IV isotherms with H_4_-type hysteresis loops, indicating the presence of mesoporous structures. The corresponding BET surface area and pore volume results (Figure 4e and Table S1) reveal dramatic improvements after metal loading. Among them, Fe/aPAC exhibits the highest surface area of 576.99 m^2^ g^–1^, followed by Ce/aPAC (473.23 m^2^ g^–1^) and Bi/aPAC (291.59 m^2^ g^–1^), which are all significantly higher than those of PAC (113.62 m^2^ g^–1^) and aPAC (34.33 m^2^ g^–1^). This observation, although seemingly contrary to the conventional understanding that metal impregnation tends to block pores and reduce the surface area, can be reasonably explained by a partial alkaline etching effect occurring during the metal loading step. The introduction of NaOH in the preparation process likely caused mild etching of the amorphous carbon matrix and removal of residual tarry substances or surface impurities, thereby reopening some previously blocked micropores and generating additional mesopores.

As a result, the specific surface area and pore volume increased notably after metal incorporation. Correspondingly, Fe/aPAC and Bi/aPAC exhibit pore volumes of approximately 0.58 cm^3^ g^–1^, while Ce/aPAC shows a similar value (0.571 cm^3^ g^–1^), all suggesting excellent capacity for gas diffusion. Furthermore, the pore size distributions (Figure 4f) derived from BJH analysis are centered around 2–3 nm, confirming the dominance of mesoporosity. Such mesoporous frameworks are particularly advantageous for gas–solid catalytic reactions as they facilitate rapid mass transfer and ensure efficient exposure of active sites within the porous network. To investigate the basic surface properties that influence H_2_S adsorption, CO_2_-TPD analysis was conducted on the metal-loaded catalysts (Figure 4g). Among the tested samples, Fe/aPAC and Ce/aPAC exhibited notably stronger CO_2_ desorption signals, indicating a higher density of surface basic sites. This enhanced basicity is attributed to the presence of metal–oxygen species and residual alkali introduced during KOH activation, both of which promote the adsorption of acidic H_2_S molecules through acid–base interactions. Moreover, the thermal stability of the materials was assessed through TGA and DTG analyses (Figure 4h,i). All samples remain stable up to ∼400 °C, indicating good thermal endurance under low-temperature catalytic conditions. Minor shifts in decomposition temperature and DTG peak positions among metal-loaded samples reflect variations in surface functional groups and the decomposition of associated inorganic components.

To further explore the surface chemical states of the catalysts, XPS analysis was performed, and the results are shown in Figure 5. The spectra provide insights into the valence states of the metal species and the bonding environments of the C and the O elements, which are critical for catalytic activity. In the Fe 2p spectrum (Figure 5a), Fe/aPAC exhibits characteristic peaks at ∼710.8 eV and ∼724.3 eV, corresponding to Fe 2p_3_/_2_ and Fe 2p_1_/_2_,^39^ respectively. The deconvolution reveals the coexistence of Fe^3+^ (Fe_2_O_3_) and FeOOH,^37^ indicating redox-active iron species on the surface, which are expected to facilitate H_2_S oxidation via Fe^3+^/Fe^2+^ cycling. The corresponding O 1s spectrum (Figure 5c) displays peaks attributable to C–O, C–OH, and lattice oxygen (Fe–O), further supporting the presence of surface oxygen species involved in redox processes. Similar observations have been reported in Fe-based biochar systems where lattice oxygen and surface hydroxyl groups contribute to the activation of H_2_S molecules.^40^

**Figure 5:**
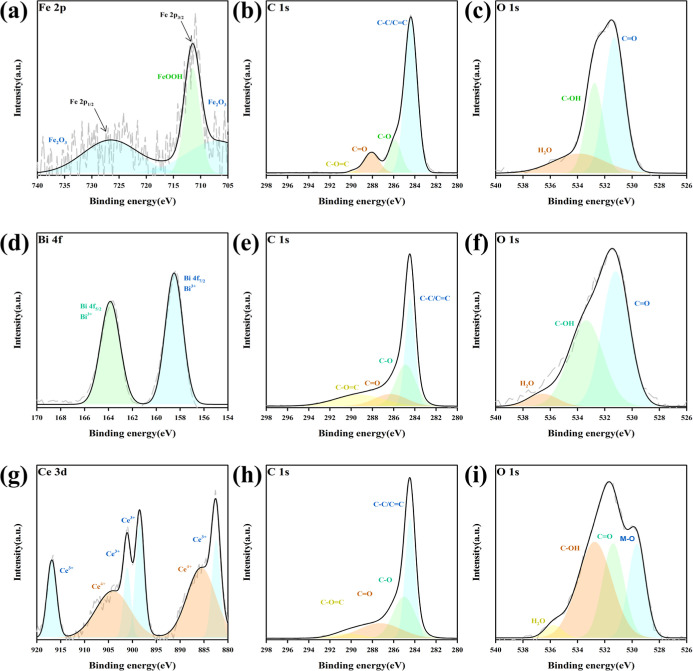
XPS spectra of (a) Fe 2p, (d) Bi 4f, and (g) Ce 3d; C 1s spectra of (b) Fe/aPAC, (e) Bi/aPAC, and (h) Ce/aPAC; and O 1s spectra of (c) Fe/aPAC, (f) Bi/aPAC, and (i) Ce/aPAC.

In Bi/aPAC, the Bi 4f spectrum (Figure 5d) shows two peaks centered at ∼159.1 eV and ∼164.4 eV, assigned to Bi 4f_7_/_2_ and Bi 4f_5_/_2_, consistent with the presence of Bi^3+^. These Bi^3+^ species may act as Lewis acid sites and contribute to the activation of H_2_S. The O 1s spectrum (Figure 5f) reveals bonding configurations similar to those of Fe/aPAC, suggesting that oxygenated carbon sites and Bi–O species coexist on the catalyst surface. For Ce/aPAC, the Ce 3d spectrum (Figure 5g) shows multiple peaks typical of mixed Ce^4+^ and Ce^3+^ oxidation states,^32,33^ confirming the redox flexibility of cerium oxide. The presence of both oxidation states enables CeO_2_ to function as an oxygen buffer, storing and releasing lattice oxygen during the reaction. This is supported by the O 1s spectrum (Figure 5i), which includes a peak assignable to M–O (Ce–O), in addition to surface hydroxyl and carbon–oxygen species.^41^ Such redox behavior has been recognized as a key factor enhancing oxygen mobility and sulfur selectivity in Ce-based catalysts.^20^

The C 1s spectra of all three catalysts (Figure 5b,e,h) exhibit three major peaks assigned to C–C/C=C (∼284.8 eV), C–O (∼286.3 eV), and O–C=O (∼288.7 eV),^41,42^ suggesting the presence of oxygen-containing surface functionalities derived from the KOH activation process. These polar groups may enhance H_2_S adsorption and facilitate surface reactions. Overall, the XPS results confirm that all three catalysts incorporate active metal species in oxidized forms (Fe^3+^, Bi^3+^, Ce^4+^/^3+^), accompanied by abundant oxygenated and hydroxyl surface groups, which together create a redox-active environment favorable for selective oxidation of H_2_S to elemental sulfur.

### 3.2 Effect of Gas Atmosphere and Catalyst Composition on H_2_S Catalytic Oxidation

The gas environment plays a critical role in determining the efficiency and selectivity of H_2_S catalytic oxidation, as it influences the availability of ROS, the redox behavior of active sites, and the stability of surface-bound sulfur species. In particular, the presence or absence of molecular oxygen and water vapor can significantly alter the reaction pathway and catalyst performance. To systematically evaluate these effects, the catalytic activities of the prepared carbon-based materials were tested under four representative conditions: dry nitrogen (N_2_), humid nitrogen (N_2_ + RH), dry air, and humid air (air + RH).^43,44^ Under all conditions, PAC and aPAC showed poor H_2_S removal capability (Figure 6a,b). In particular, aPAC exhibited a rapid initial conversion that quickly declined regardless of the gas environment, indicating limited reactive sites and low oxidative potential. However, upon metal loading, the catalytic activity improved dramatically, especially, Fe/aPAC (Figure 6c) achieved nearly complete H_2_S conversion within 5 min under all conditions and sustained high efficiency throughout the 60 min test, particularly in humid air. The superior performance can be attributed to the presence of Fe^3+^ species (Fe_2_O_3_ and FeOOH), which are capable of activating molecular oxygen and facilitating redox cycling between Fe^3+^ and Fe^2+^, thereby enabling the continuous catalytic oxidation of H_2_S to elemental sulfur.

**Figure 6:**
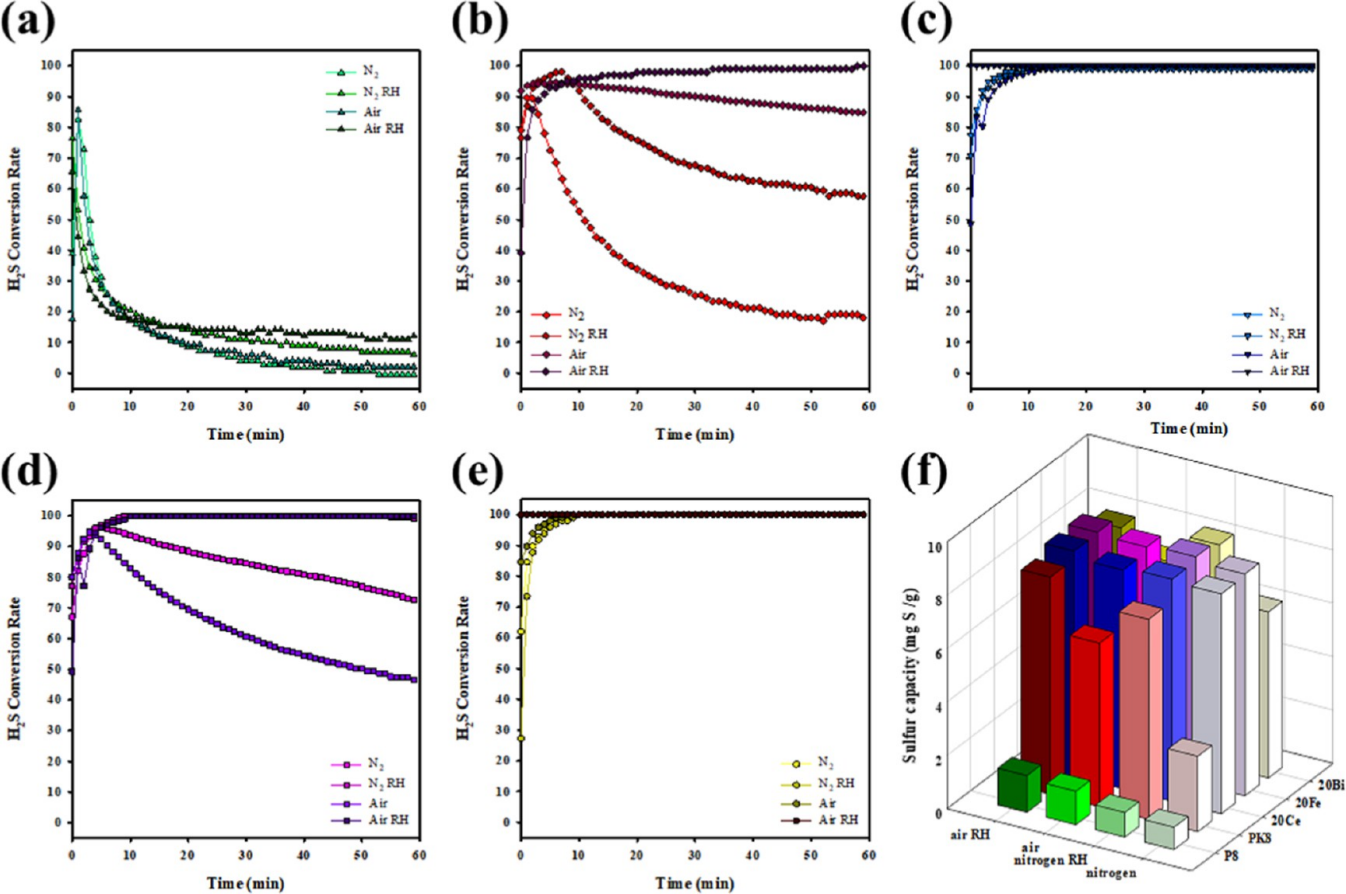
H_2_S conversion profiles of (a) PAC, (b) aPAC, (c) Fe/aPAC, (d) Bi/aPAC, and (e) Ce/aPAC under different gas environments (N_2_, air, RH, and Air); (f) comparison of sulfur capacity under each condition ([H_2_S] = 100 ppm, temperature = 30 °C, catalyst = 0.2 g, total flow rate = 200 mL/min).

Bi/aPAC (Figure 6d) also demonstrated high activity under oxidative atmospheres, especially in air and air + RH, although its performance was slightly inferior to Fe/aPAC in terms of long-term stability. The Bi^3+^ species on the surface likely contribute to oxidation via Lewis acid catalysis and oxygen transfer mechanisms; however, their redox versatility is lower compared to Fe. Among the three catalysts, Ce/aPAC (Figure 6e) exhibited the most stable and consistent catalytic behavior under all gas environments, including N_2_, owing to the unique oxygen storage-release capability of Ce^4+^/Ce^3+^ redox pairs. Its performance remained near 100% conversion over 60 min, even in the absence of molecular oxygen, indicating that lattice oxygen from CeO_2_ actively participated in the oxidation of H_2_S.

Sulfur capacity measurements (Figure 6f) further support these observations. Ce/aPAC and Fe/aPAC both achieved the highest sulfur yields (∼9 mg S/g), followed by Bi/aPAC (∼8 mg S/g), with PAC and aPAC showing minimal sulfur formation. Notably, humid air conditions led to enhanced sulfur capacity for all active catalysts, highlighting the promotional role of water vapor in facilitating surface reactions and preventing sulfur poisoning.

### 3.3 Effect of Gas Flow Rate on Catalytic Performance of H_2_S Oxidation

In a continuous-flow catalytic system, the gas flow rate directly governs the GHSV, which determines the residence time of the reactants in contact with the catalyst. As such, flow rate is a critical parameter influencing the conversion efficiency and sulfur yield in H_2_S catalytic oxidation. High flow rates may reduce contact time, leading to incomplete conversion, while excessively low flow may increase mass transfer resistance or cause sulfur accumulation on the surface. Therefore, understanding the flow-dependent behavior is essential for evaluating catalyst robustness under practical conditions.

To explore this, the catalytic performance of PAC, aPAC, Fe/aPAC, Bi/aPAC, and Ce/aPAC was tested under three flow rates: 200, 400, and 600 mL/min. As previously observed, PAC and aPAC (Figure 7a,b) displayed a limited H_2_S removal ability across all flow conditions. The rapid drop in conversion, even at the lowest flow rate, indicates insufficient active sites and a poor interaction between H_2_S and the carbon surface. Among the metal-loaded samples, Fe/aPAC (Figure 7c) demonstrated the most balanced and effective catalytic behavior. At 200 mL/min, it achieved nearly complete H_2_S conversion, maintaining a greater than 95% activity throughout the test. Even at 400 mL/min, its conversion remained high with only a minor decline over time. While a more noticeable decrease was observed at 600 mL/min, Fe/aPAC still outperformed the other materials under the same condition. Its sulfur capacity (Figure 7f) also remained consistently high, especially under moderate flow, indicating its excellent ability to oxidize and store sulfur species without premature deactivation. The strong performance of Fe/aPAC can be attributed to the redox-active Fe^3+^/Fe^2+^ species and the synergistic interaction with the porous carbon matrix, which facilitate both oxygen activation and H_2_S adsorption.

**Figure 7:**
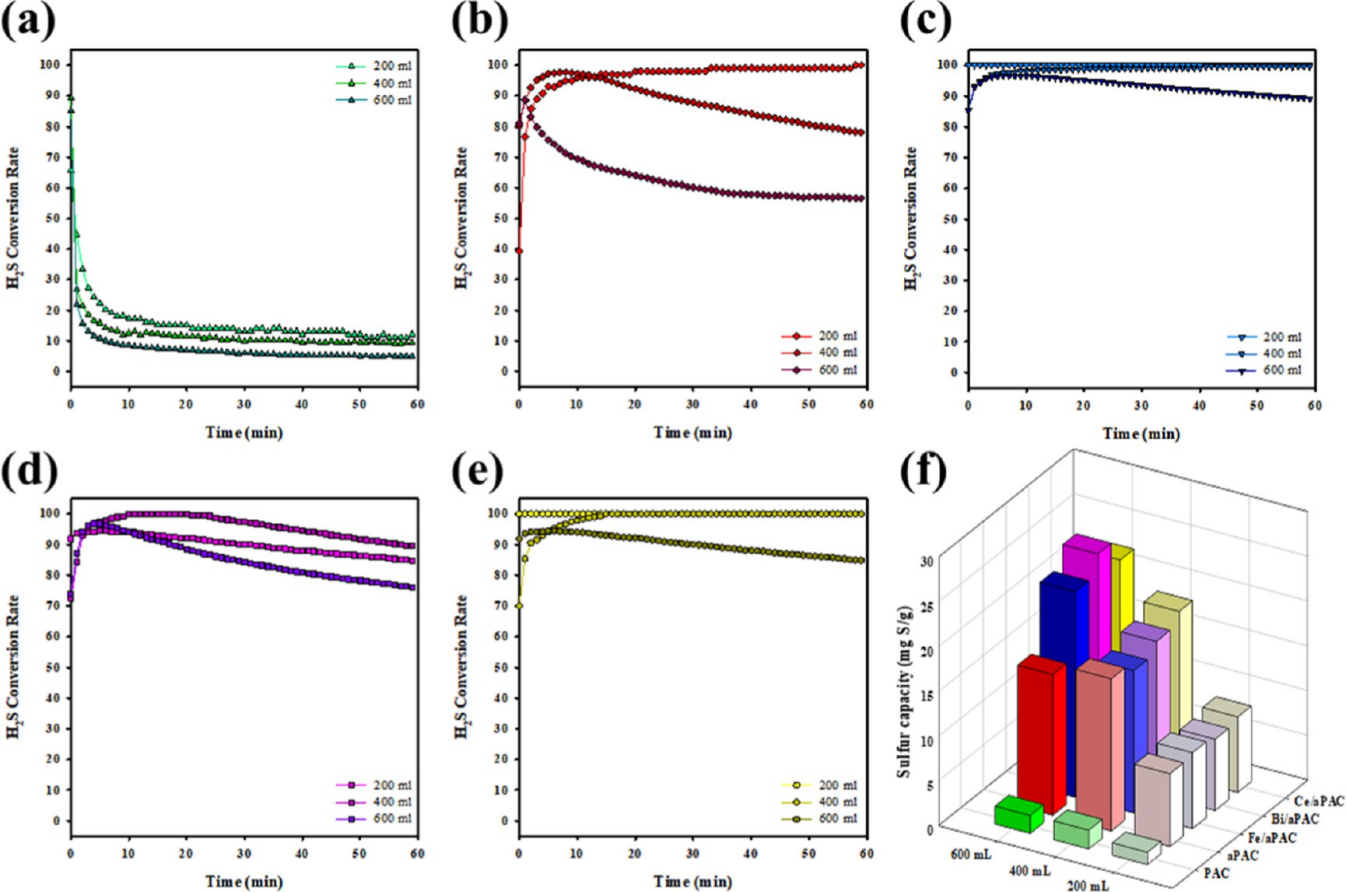
H_2_S conversion profiles of (a) PAC, (b) aPAC, (c) Fe/aPAC, (d) Bi/aPAC, and (e) Ce/aPAC under different total flow rates (200, 400, and 600 mL/min); (f) comparison of sulfur capacity under each flow condition. ([H_2_S] = 100 ppm, temperature = 30 °C, catalyst = 0.2 g, total flow rate = 200–600 mL/min, humidified with room-temperature saturated water vapor).

Bi/aPAC (Figure 7d) exhibited a moderate sensitivity to flow variation. Its conversion remained relatively stable under 200 and 400 mL/min but decreased more noticeably at 600 mL/min. The reduction in performance may be due to limited oxygen activation capacity or slower redox cycling of the Bi^3+^ species under shorter contact times. On the other hand, Ce/aPAC (Figure 7e) showed remarkable stability in conversion across all flow rates, likely due to the oxygen storage-release behavior of CeO_2_. However, its sulfur capacity decreased more sharply with increasing flow (Figure 7f), suggesting that although Ce-based materials maintain conversion, the extent of full oxidation to solid sulfur may be incomplete under high GHSV, possibly due to kinetic limitations in lattice oxygen replenishment.

### 3.4 Effect of Reaction Temperature on Catalytic Performance

Reaction temperature is a key factor influencing the kinetics of H_2_S catalytic oxidation, especially under low-temperature conditions. To evaluate the thermal sensitivity of the catalysts, Fe/aPAC, Bi/aPAC, and Ce/aPAC were tested at 30 °C, 40 °C, and 50 °C under room-temperature saturated water vapor conditions. The corresponding H_2_S conversion profiles and sulfur capacities are shown in Figure 8. Among the three catalysts, Fe/aPAC (Figure 8a) exhibited the most noticeable temperature-dependent enhancement. At 30 °C, the conversion rate was already above 90% and remained stable throughout the test. With increasing temperature to 40 and 50 °C, not only was the initial conversion slightly improved but the long-term stability and total sulfur capacity also increased (Figure 8d). This trend suggests that Fe-based active sites benefit from mild thermal activation, which facilitates redox cycling between Fe^3+^ and Fe^2+^, enhancing the continuous oxidation of H_2_S to elemental sulfur.

**Figure 8:**
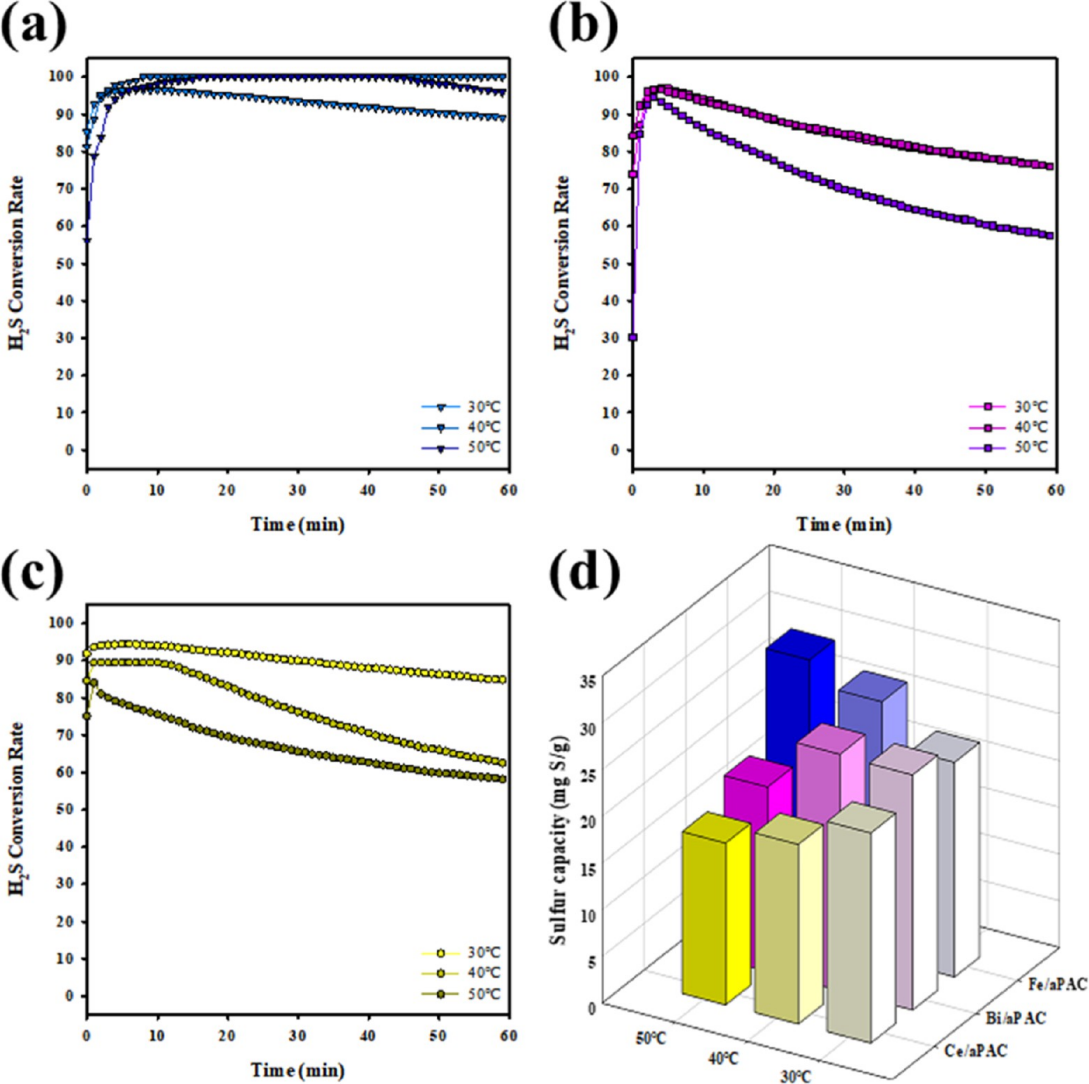
Effect of temperature on H_2_S conversion over (a) Fe/aPAC, (b) Bi/aPAC, and (c) Ce/aPAC at 30, 40, and 50 °C under humid conditions (RT saturated vapor); (d) comparison of sulfur capacity ([H_2_S] = 100 ppm, catalyst = 0.2 g, flow rate = 600 mL/min).

Bi/aPAC (Figure 8b) showed moderate activity at all of the tested temperatures. Although the initial H_2_S conversion exceeded 90% across the range, the decline in conversion became more apparent at 30 and 40 °C. Raising the temperature to 50 °C improved the stability slightly, resulting in a modest increase in sulfur capacity. This indicates that Bi^3+^ sites may require elevated temperature to sustain their oxidative potential, possibly due to slower surface oxygen exchange kinetics compared to Fe. In contrast, Ce/aPAC (Figure 8c) displayed stable performance across all temperatures but with a less pronounced response to thermal variation. Its conversion remained consistently above 90%, but the sulfur capacity (Figure 8d) did not increase significantly with the temperature. This behavior is attributed to the intrinsic oxygen storage-release property of CeO_2_, which provides lattice oxygen for oxidation regardless of small temperature changes. However, at higher temperatures, CeO_2_ may favor partial oxidation pathways (e.g., formation of SO_2_) or faster desorption of intermediates, leading to a plateau in sulfur accumulation.

In addition to the temperature, the catalytic performance of H_2_S oxidation is also influenced by environmental factors such as humidity, reaction duration, and the reusability of the catalyst. These aspects were further examined to evaluate the practical stability and regeneration ability of the prepared catalysts. The influence of the humidity on H_2_S oxidation over Fe/aPAC is shown in Figure S3. All curves rapidly reach over 90% conversion and remain stable during the test. A slight increase in activity with rising humidity (30–50% RH) indicates that moderate water vapor facilitates H_2_S oxidation, likely by generating surface hydroxyl groups and enhancing oxygen mobility. Within this range, Fe/aPAC maintains high conversion and stability, confirming its good water tolerance under humid conditions.

### 3.5 The Proposed Mechanism for Catalytic Oxidation of H_2_S to S Using Fe/aPAC

To clarify the mechanism underlying H_2_S catalytic oxidation, postreaction Fe/aPAC was analyzed using XRD, Raman, FTIR, SEM-EDS, elemental mapping, and XPS (Figures 9 and S2). The XRD pattern (Figure 9a) of used Fe/aPAC reveals characteristic peaks attributed to FeS and S_8_, indicating the formation of sulfur species on the catalyst surface.^45^ This is further supported by the Raman spectrum (Figure 9b), where signals corresponding to S–S and Fe–S vibrations are evident. FTIR results (Figure 9c) also confirm the presence of Fe–S, SO_4_^2–^, and C–S functionalities, suggesting that both elemental sulfur and oxidized sulfur species were formed during the reaction. XPS analysis (Figure S2) provides detailed insight into the chemical states. In the Fe 2p spectrum (Figure S2a), both the Fe^2+^ and Fe^3+^ states are identified, suggesting a redox cycle between Fe^3+^ and Fe^2+^ during catalysis. The S 2p spectrum (Figure S2b) shows peaks for S^0^, S^6+^, and FeS,^46,47^ confirming the coexistence of various sulfur species. Moreover, the C 1s and O 1s spectra (Figure S2c,d) demonstrate the presence of oxygenated groups (C=O, C–OH, and adsorbed H_2_O), which can enhance surface reactivity and facilitate H_2_S adsorption and oxidation. SEM/EDS (Figure 9d,e) and elemental mapping (Figure 9f–i) verify the uniform distribution of Fe, S, O, and C, indicating well-dispersed iron sites and effective sulfur deposition. The sulfur content (4.18 wt %) also corroborates efficient H_2_S conversion and accumulation of reaction products. The reusability of Fe/aPAC was evaluated through three consecutive H_2_S oxidation cycles, as shown in Figure S4. After each cycle, the used catalyst was regenerated by extracting the deposited sulfur with toluene under ultrasonic agitation for 30 min, followed by drying at 80 °C. The catalyst maintained nearly 100% H_2_S conversion in the first cycle, which slightly decreased to ∼90% and ∼80% in the second and third cycles, respectively. The gradual decline in activity can be attributed to residual sulfur or sulfate species partially blocking active sites after repeated use. Nevertheless, Fe/aPAC retained over 80% of its original performance after three cycles, demonstrating good structural stability and regeneration potential. In addition, extracted elemental sulfur was visibly collected after toluene washing (Figure S5), providing further evidence of S^0^ formation and recoverability. Moreover, a comparison of the H_2_S removal capacity with previously reported carbon-based or metal oxide-based catalysts is summarized in Table S2. As shown, Fe/aPAC exhibits a high sulfur capacity of 33.5 mg of S/g, outperforming many literature-reported catalysts under similar conditions. This superior performance, combined with excellent reusability and a recoverable sulfur product, highlights the practical potential of Fe/aPAC for low-temperature desulfurization applications.

**Figure 9:**
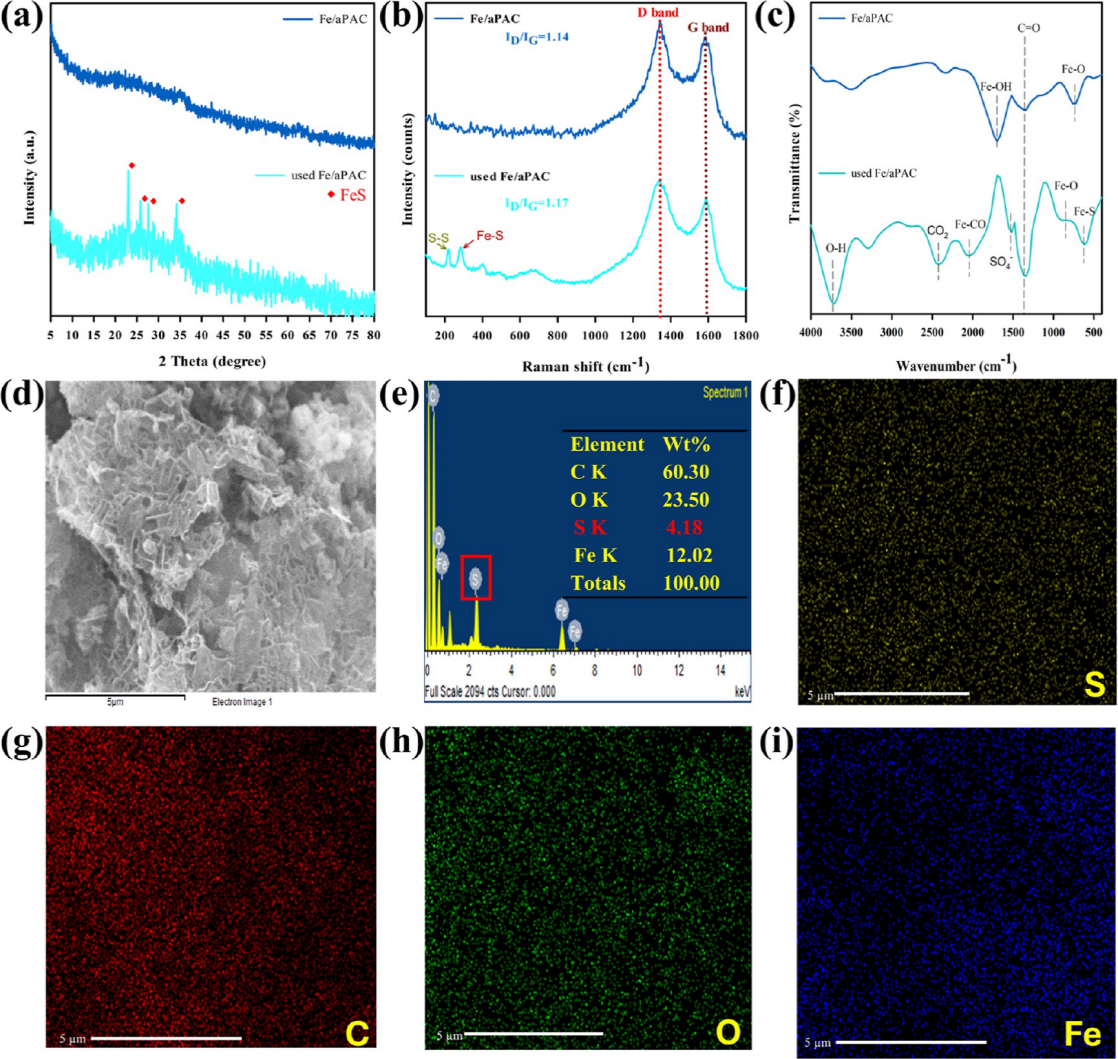
(a) XRD pattern, (b) Raman spectra, (c) FTIR spectra, (d) SEM image, (e) EDS spectrum, and (f–i) elemental mapping of S, C, O, and Fe for used Fe/aPAC.

This dual-pathway mechanism—comprising both direct oxidation via oxygen and Fe-mediated redox reactions—ensures high efficiency for H_2_S removal and selective S formation under mild conditions. Based on these results, a plausible mechanism is proposed (Figure 10). Initially, H_2_S is adsorbed onto the catalyst surface and dissociates into HS^–^ and H^+^ on the catalyst surface.^48^ Simultaneously, O_2_ molecules are activated on the surface or via Fe^3+^-mediated redox reactions.^49^ The HS^–^ intermediate is then oxidized by either lattice oxygen (O^2–^) or surface-adsorbed oxygen species,^50,51^ forming S^0^ and H_2_O. The redox cycle of Fe^3+^/Fe^2+^ facilitates continuous electron transfer and regeneration of active sites,^52^ as represented by eqs 2–4

3 $${Fe}^{3+}+{HS}^{-}\to {Fe}^{2+}+S^{0}+H^{+}$$

4 $${Fe}^{2+}+{1/2O}_{2}\to {Fe}^{3+}+O^{2-}$$

in addition, under prolonged oxidation or high O_2_ availability, part of the surface S^0^ can undergo further oxidation through lattice oxygen to form sulfate species (SO_4_^2–^)­

5 $$S^{0}+{2O}_{2}\to {\text{SO}}_{4}^{2-}$$

**Figure 10:**
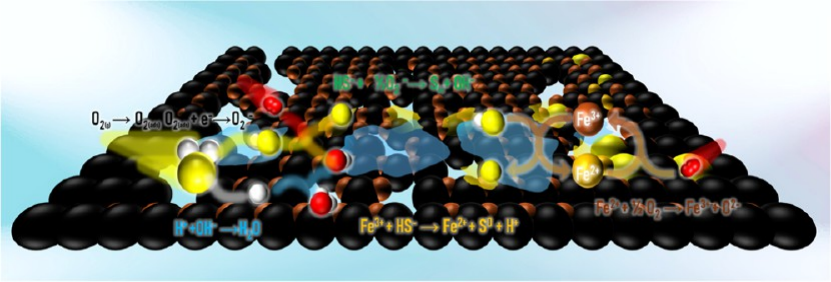
Proposed mechanism of H_2_S catalytic oxidation to elemental sulfur over Fe/aPAC under humid conditions.

This overoxidation step explains the SO_4_^2–^ signals observed in both XPS (S 2p) and FTIR spectra of the used catalyst, indicating that a fraction of sulfur species was transformed into stable surface-bound sulfate (M–O–SO_3_/SO_4_) complexes during the reaction.

This dual-pathway mechanism—comprising both direct oxidation via oxygen and Fe-mediated redox reactions—ensures high efficiency for H_2_S removal and selective S formation under mild conditions.

## 4 Conclusions

A series of single-metal-supported catalysts (Fe, Bi, and Ce) were synthesized on PET-derived activated carbon (aPAC) to evaluate their performance in H_2_S catalytic oxidation under environmentally relevant conditions. Among them, Fe/aPAC exhibited the most outstanding catalytic activity, achieving over 90% H_2_S conversion and a high sulfur yield at 30 °C under humid air, which can be attributed to its favorable surface properties and redox behavior. Detailed characterization revealed that the superior performance of Fe/aPAC was associated with the presence of Fe_2_O_3_/FeOOH species, abundant surface oxygenated groups (C–O, C=O, O–H), and the formation of FeS and S^0^ after reaction, as evidenced by XPS, FTIR, and XRD analyses. The presence of water vapor enhanced surface hydroxyl formation and facilitated Fe^3+^/Fe^2+^ redox cycling, contributing to the improved H_2_S oxidation efficiency and sulfur selectivity. A plausible reaction pathway was proposed, involving dissociative adsorption of H_2_S, Fe^3+^-mediated oxidation to elemental sulfur, and regeneration of Fe^3+^ via O_2_ activation. This study provides mechanistic insights into low-temperature H_2_S oxidation and demonstrates the feasibility of upcycling plastic waste into functional catalysts for environmental remediation. The proposed approach offers a sustainable strategy that integrates plastic waste management with air pollution control, in line with the principles of green chemistry and circular economy.

## Supplementary Material



## Data Availability

All data supporting the findings of this study are included in the published article and its Supporting Information. And data can be provided by the corresponding author upon reasonable request.

## Notes

Experimental details; CO_2_-TPD and sulfur extraction methods; additional XPS and EDS data of used catalysts; humidity and reusability test results; and supplementary figures and tables (PDF)
