# Supplementary material for: Selective Oxidation of H2S to Elemental Sulfur over Waste PET-Derived Biochar-Supported Metal Catalysts
Source: ACS Omega. 2025 Dec 8;10(50):61828–40. doi: 10.1021/acsomega.5c08359 (PMC12750196; doi:10.1021/acsomega.5c08359)
Supplement: Supplementary file 1 [file ao5c08359_si_001.pdf]

Supplementary Material

For

Selective Oxidation of H_2S to Elemental Sulfur over Waste PET-Derived Biochar Supported Metal Catalysts

Jia-Yin Lin[abc], Guan-Jie Wang[a], Jia-Yu Lee[a], Yu-Lun Wu[b] and Chih-Ying Wang[a]*

[a] Graduate Program in Semiconductor and Green Technology, Academy of Circular Economy, National Chung Hsing University, Taichung 402, Taiwan

[b] Graduate Program in Industrial and Smart Technology, Academy of Circular Economy, National Chung Hsing University, Taichung 402, Taiwan

[c] Innovation and Development Center of Sustainable Agriculture, National Chung Hsing University, Taichung 402, Taiwan

*Corresponding Authors. E-mail addresses: joylin7@dragon.nchu.edu.tw (*Jia-Yin Lin*)

S1. The CO_2 temperature-programmed desorption

To evaluate the surface basicity of the catalysts, CO_2 temperature-programmed desorption (CO_2-TPD) analysis was performed using a chemisorption analyzer (ASIQ TPx, Anton Paar, Austria). Prior to CO_2 adsorption, the samples underwent thermal pretreatment at 400 °C for 1 hour to remove any physisorbed species. After cooling to room temperature (25 °C), the catalyst was exposed to a 5% CO_2/N_2 gas mixture at a flow rate of 20 mL min^{-1} for 1 hour to allow CO_2 adsorption. Subsequently, the system was purged with pure N_2 while the temperature was ramped from 25 °C to 800 °C at a rate of 10 °C min^{-1} to desorb the adsorbed CO_2 species.

S2. Extraction of Sulfur from Spent Activated Carbon Catalysts

To recover the deposited sulfur species from the used activated carbon catalysts after H_2S oxidation, a simple solvent extraction method was employed. Briefly, the used catalyst sample was immersed in toluene and stirred gently at room temperature for 24 h to dissolve elemental sulfur. After extraction, the suspension was filtered through qualitative filter paper to remove the solid residue. The filtrate was then evaporated, and the recovered yellow solid was dried in an oven at 60 °C to obtain elemental sulfur (S^0).

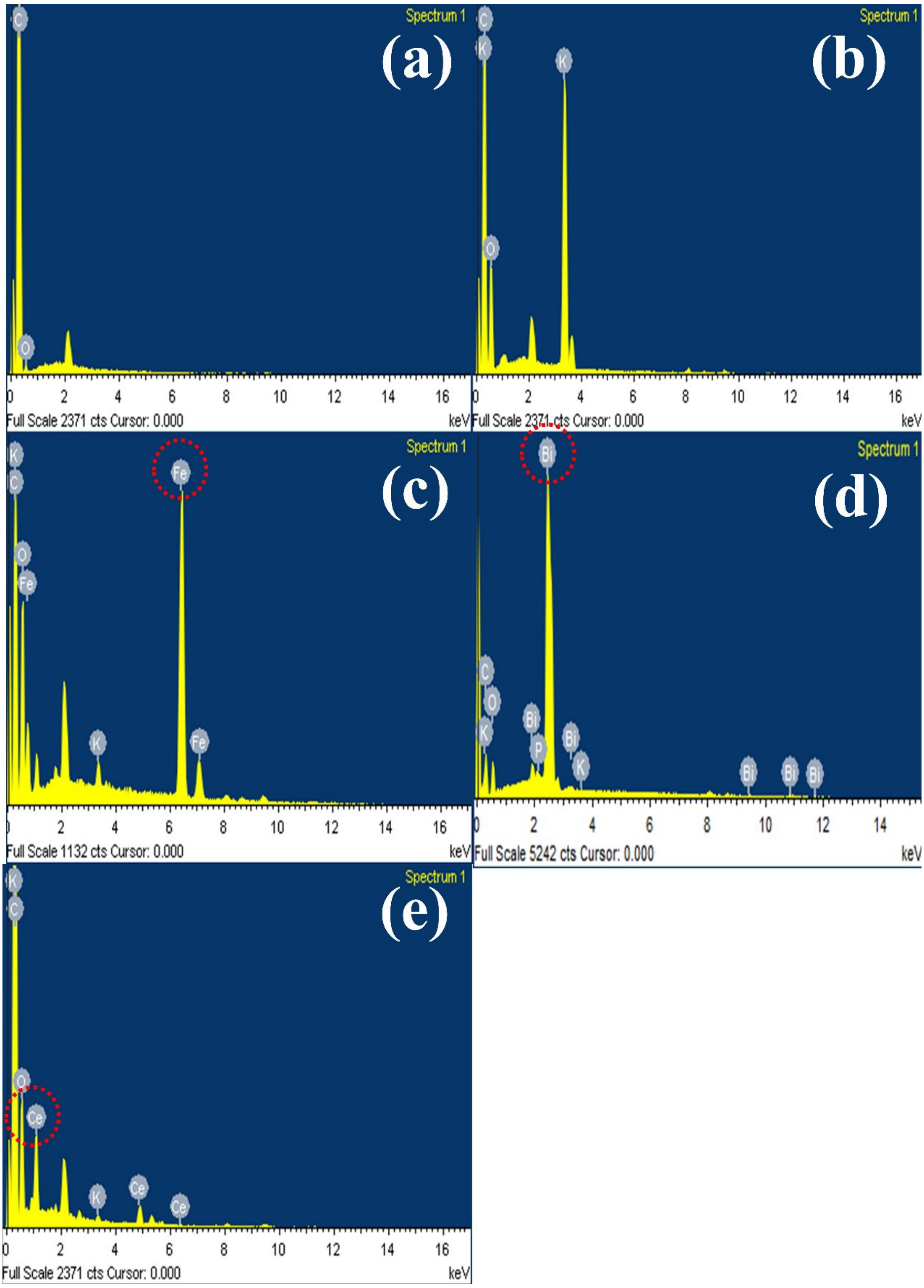

Figure S1. EDS spectra of (a)PAC, (b)aPAC,(c)Fe/aPAC ,(d)Bi/aPAC ,and (e)Ce/aPAC

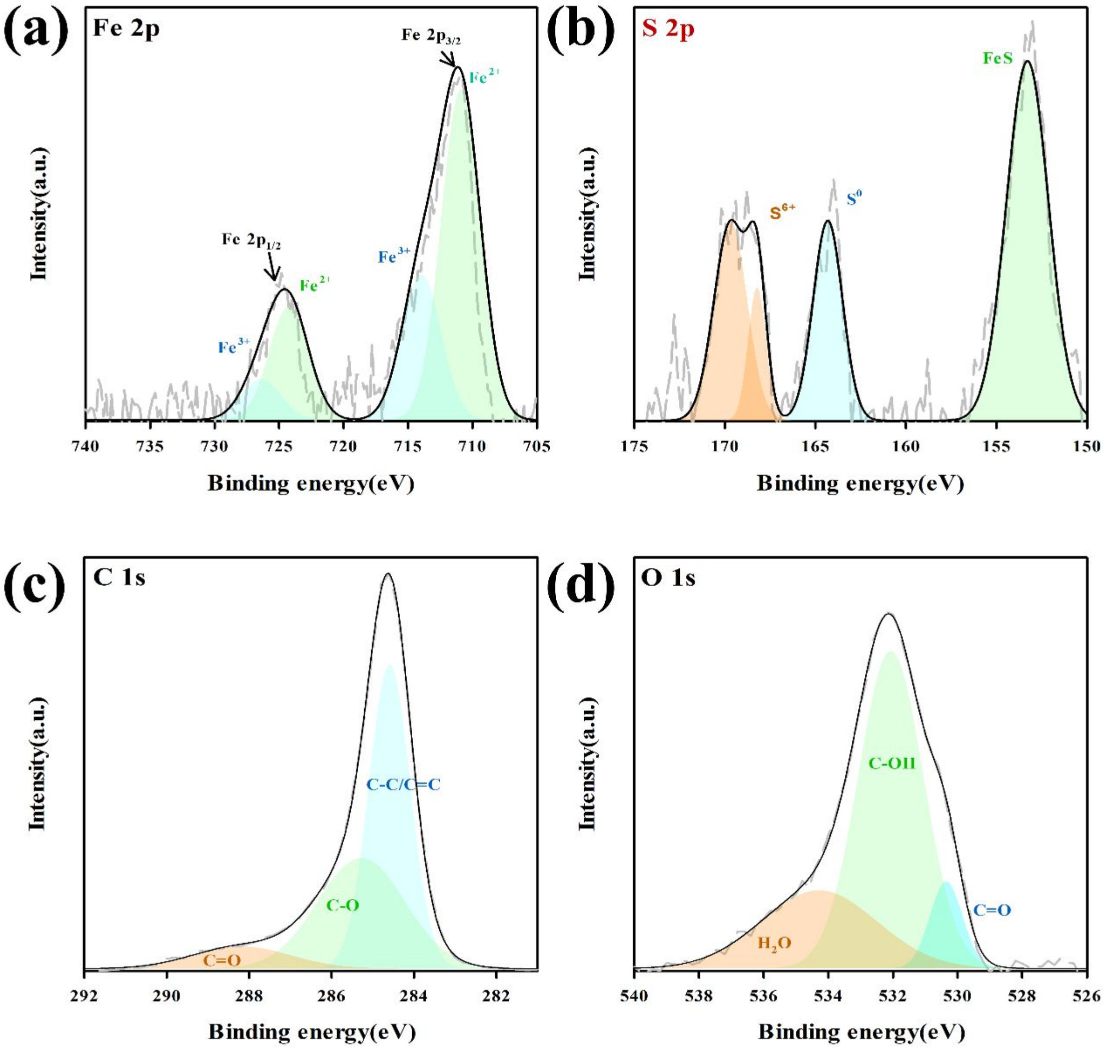

Figure S2. XPS spectra of used Fe/aPAC: (a) Fe 2p, showing the presence of Fe^{2+} and Fe^{3+} species; (b) S 2p, indicating the coexistence of elemental sulfur (S^0), sulfate (S^{6+}), and iron sulfide (FeS); (c) C 1s, revealing carbon–carbon, C–O, and C=O functional groups; and (d) O 1s, deconvoluted into C–OH, H_2O, and C=O components.

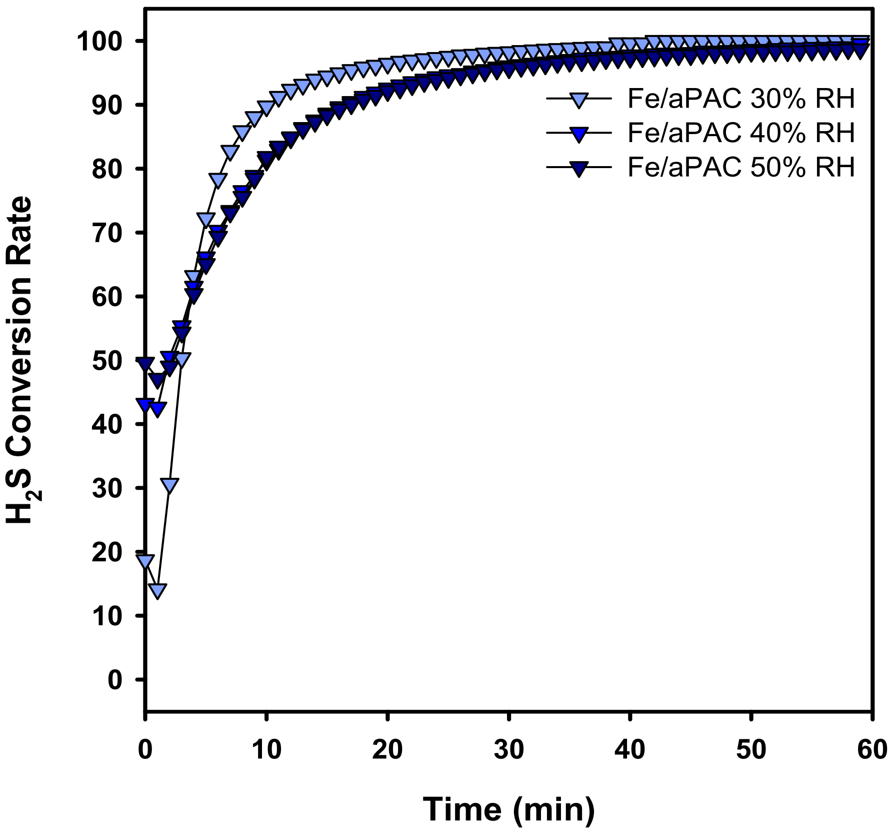

Figure S3. H_2S conversion performance of Fe/aPAC under different relative humidity conditions (30%, 40%, and 50% RH). Reaction conditions: $[H_2S]$ = 100 ppm, Temperature = 50 °C, Catalyst loading = 0.2 g, Total gas flow rate = 200 mL min^{-1}.

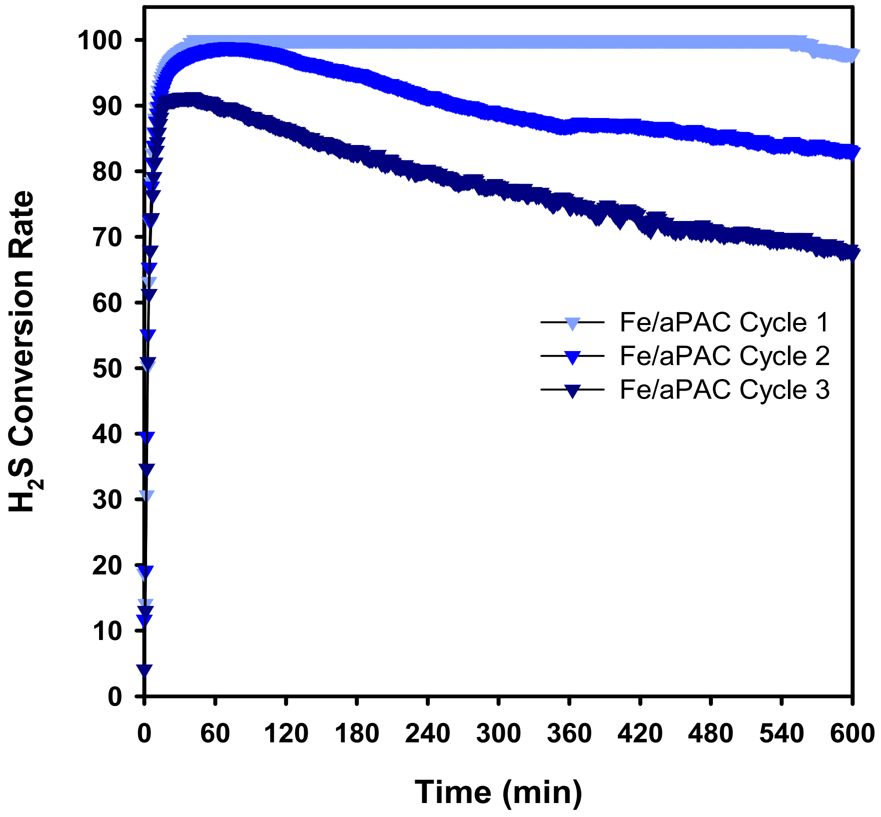

Figure S4. Long-term H_2S conversion performance of Fe/aPAC over three consecutive cycles. Reaction conditions: $[H_2S]$ = 100 ppm, Temperature = 50 °C, Catalyst loading = 0.2 g, Total gas flow rate = 200 mL min^{-1}.

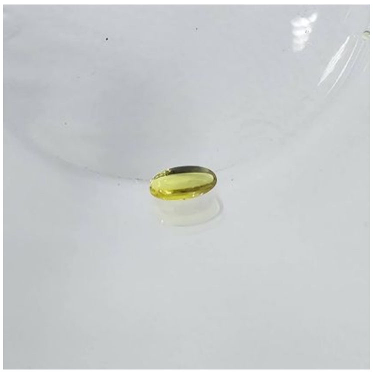

Figure S5. Photograph of extracted elemental sulfur after toluene extraction from used Fe/aPAC catalyst. The sulfur was recovered by soaking the spent catalyst in toluene for 24 h, followed by filtration and drying, resulting in visible S^0 precipitation at the bottom of the vial.

Table S1. Textural properties of PAC, aPAC, and metal-loaded aPAC catalysts.

Sample	Surface area (m^2/g)	Pore Volume (cc/g)
PAC	113.615	0.000
aPAC	34.329	0.097
Fe/aPAC	576.994	0.582
Bi/aPAC	291.594	0.167
Ce/aPAC	473.232	0.571

Table S2. H_2S removal capabilities of other materials

Sample	H_2S remove capacity (mg S /g)	Reference
Fe/aPAC	33.5	This work
HCK-220–6	21	[1]
ZnBW	12.5	[2]
$Cu_{0.5}Zn_{0.5}$/AC	27.2	[3]
ZAN-0-20	30.5	[4]

Reference

(1) Chen, W.; Zhang, G.; Li, D.; Ma, S.; Wang, B.; Jiang, X. Preparation of nitrogen-doped porous carbon from waste polyurethane foam by hydrothermal carbonization for H2S adsorption. *Industrial & Engineering Chemistry Research* **2020**, *59* (16), 7447-7456.

(2) Nguyen-Thanh, D.; Bandosz, T. J. Activated carbons with metal containing bentonite binders as adsorbents of hydrogen sulfide. *Carbon* **2005**, *43* (2), 359-367.

(3) Balsamo, M.; Cimino, S.; De Falco, G.; Erto, A.; Lisi, L. ZnO-CuO supported on activated carbon for H2S removal at room temperature. *Chemical Engineering Journal* **2016**, *304*, 399-407.

(4) Yang, C.; Yang, S.; Fan, H.; Wang, Y.; Shangguan, J. Tuning the ZnO-activated carbon interaction through nitrogen modification for enhancing the H2S removal capacity. *Journal of Colloid and Interface Science* **2019**, *555*, 548-557.
